# Encapsulation of Benzaldehyde Produced by the Eco-Friendly Degradation of Amygdalin in the Apricot Kernel Debitterizing Wastewater

**DOI:** 10.3390/foods13030437

**Published:** 2024-01-29

**Authors:** Lei Song, Juan Francisco García Martín, Qing-An Zhang

**Affiliations:** 1 School of Food Engineering and Nutrition Science, Shaanxi Normal University, Xi’an 710119, China; 2 Departamento de Ingeniería Química, Facultad de Química, Universidad de Sevilla, s/n, 41012 Seville, Spain; jfgarmar@us.es

**Keywords:** benzaldehyde, eco-friendly preparation, amygdalin, encapsulation, molecular simulation

## Abstract

In order to fully utilize the by-products of apricot kernel-debitterizing and address the chemical instability of benzaldehyde in the food industry, benzaldehyde was first prepared by adding the apricot kernel powder to degrade the amygdalin present in the apricot kernel-debitterizing water. Subsequently, β-cyclodextrin was employed to encapsulate the benzaldehyde, and its encapsulation efficacy was evaluated through various techniques including Fourier transform infrared spectroscopy, thermogravimetric analysis, release kinetics fitting inhibitory effect and the effect on *Botrytis cinerea*. Finally, the encapsulation was explored via molecular docking and molecular dynamics simulations. The results indicate that the optimal preparation conditions for the benzaldehyde were 1.8 h, 53 °C and pH 5.8, and the encapsulation of benzaldehyde with β-cyclodextrin (wall–core ratio of 5:1, mL/g) has been verified by the deceleration in the release rate, the enhanced thermal stability and the prolonged inhibition effect against *Botrytis cinerea*. The encapsulation proceeded spontaneously without steric hindrance in the simulation, which led to a reduction in the hydrophobic cavity of β-cyclodextrin. In conclusion, the amygdalin in the debitterizing wastewater can be degraded in an eco-friendly way to produce benzaldehyde by adding apricot kernel powder, which contains β-glucosidase; the encapsulation of benzaldehyde is stable, thus enhancing the utilization of amygdalin in the debitterizing wastewater of apricot kernels.

## 1. Introduction

Amygdalin, a cyanogenic glucoside, can hydrolyze into hydrogen cyanide (HCN) in the body, which is a potential toxic substance abundantly presented in apricot kernels [1]. Thus, the removal of amygdalin (debitterizing) was a necessary procedure in apricot kernel processing [2]. A large amount of amygdalin was transferred to the debitterizing wastewater, so the direct discharge of such wastewater would cause environmental pollution and the waste of resources [3,4]. According to preliminary research, Amygdalin can be degraded into benzaldehyde by β-glucosidase, making the apricot kernel debitterizing wastewater a potential raw material for use in benzaldehyde production [5].

Due to its non-toxic nature, ease of accessibility, and possession of antibacterial and antioxidant properties, benzaldehyde demonstrated substantial potential for applications in the food industry. In addition, research has shown that benzaldehyde is an important substance for use in anti-corrosion and mosquito-repellent essential oils [6,7,8]. The most commonly adopted environmentally friendly method for preparing benzaldehyde utilizes natural cinnamon oil or cinnamaldehyde as raw materials, but these methods had drawbacks such as requiring expensive catalysts, creating harsh reaction conditions, and showing susceptibility to reaction failure [9]. Compared with the most commonly adopted method, utilizing amygdalin in debitterizing wastewater as a raw material has many advantages, such as lower cost, the easy availability of the catalyst, the mild reaction conditions and the simple process. Therefore, it is of great value to research the process of degrading amygdalin in debittering wastewater into benzaldehyde and extracting it. However, the direct addition of benzaldehyde to food has various drawbacks, including impacting the food’s taste due to its apricot kernel flavor, quick loss through rapid volatilization, and transforming into benzoic acid under the influence of ultraviolet light and oxygen.

To enhance the application of benzaldehyde in the food industry, an encapsulation technology needs to be developed to reduce its volatile degradation and control its release rate. As a frequently utilized encapsulation material, β-cyclodextrin can employ guest molecules to cover the fragrance, slow down the release rate and improve the stability [10,11]. Szejtli explained that β-CD exhibited a degree of hydrophobicity due to its high electron cloud density within the cavity.; the secondary hydroxyl group on the surface of cyclodextrin makes its large opening end and outer wall hydrophilic. This unique characteristic of β-CD makes it a versatile host for various guest molecules, including organic compounds, inorganic ions, and inert gases, allowing it to enhance their stability and enable controlled release [12]. Furthermore, Rukmani’s research confirmed that β-CD encapsulation is among the most effective methods to protect active compounds from oxidation, thermal degradation and evaporation, and it also serves as an efficient approach for masking undesired odors or flavors while enhancing solubility [13]. These examples have demonstrated that the encapsulation of benzaldehyde with β-cyclodextrin is a viable solution to address the issues associated with benzaldehyde. Despite research focusing on the encapsulation of β-cyclodextrin as a natural preservative for food processing applications, no work has been done on the effects and mechanisms of the β-cyclodextrin encapsulation of benzaldehyde. Furthermore, there has been a lack of research on the production of safe and available benzaldehyde through the application of β-glucosidase for amygdalin treatment in apricot kernel-debitterizing wastewater.

In enhancing the added value of apricot kernel processing, this study did not directly recover amygdalin from debitterizing wastewater, but it innovatively transformed it into benzaldehyde, a compound with greater market demand and wider applications. In terms of bitter almond degradation methods, this study avoided costly microbial degradation, opting for the utilization of the complex enzymes present in the apricot kernel to degrade the debitterizing wastewater. In this research, the apricot kernel-debitterizing wastewater was utilized as a raw material to produce benzaldehyde, and the associated parameters were optimized. Subsequently, β-cyclodextrin was employed to encapsulate benzaldehyde; then, the encapsulation effect was evaluated by various means, and the encapsulation mechanism has been explored via molecular simulations. The main purpose of this research was to systematically explore the feasibility of using amygdalin in the debitterizing wastewater to prepare benzaldehyde in an environment-friendly way, and using β-cyclodextrin to encapsulate benzaldehyde in order to enhance the added value of apricot kernels in the processing industry, as well as solving the problems of the instability and volatility of benzaldehyde.

## 2. Materials and Methods

### 2.1. Materials and Reagents

Apricot kernels were purchased from the Northwest Herb Market of Xi’an, Shaanxi Province, China. With the impurities removed, the seeds were soaked in boiling water for several minutes, and then manually peeled. Then, 5 kg of the peeled apricot kernels were put in a steam-jacked kettle with 50 L water (material to liquid ratio of 1:10, kg:L) for debitterizing at the temperature of 65 to 75 °C for 6 h, and the debitterizing wastewater was collected and stored at 4 °C for further use.

Benzaldehyde standard was purchased from Macklin Company (Shanghai, China). β-CD was purchased from LookChem Company (Zhejiang, China). Methanol of high-performance liquid chromatography (HPLC) purity was purchased from OceanPak Company (Tianjin, China).

### 2.2. Preparation of Benzaldehyde

The apricot kernel powder was added to the debitterizing wastewater at to the ratio (material to liquid) of 1:125 (g/mL), and then the pH was adjusted. Subsequently, the reaction was carried out at a certain temperature. The reaction product underwent steam distillation, followed by extraction with 80 mL of n-hexane and subsequent pressure concentration, resulting in the isolation of benzaldehyde.

### 2.3. Determination of Benzaldehyde Amygdalin and Hydrocyanic Acid Content

Determination of benzaldehyde: An isocratic elution was applied for the separation of benzaldehyde with the mobile phase of methanol-H_2_O (35:65; *v*/*v*), a 1.0 mL/min flow rate, a 35 °C column temperature, a 250 nm detection wavelength and a 20 mL injection volume [14]. The standard curve was y = 1345.2442x − 0.6348 (R^2^ = 0.9998), and the content of benzaldehyde was calculated

Determination of amygdalin: We referred to Zhang’s method and made slight modifications. The HPLC working parameters were as follows: a sample injection volume of 20 mL, a 35 °C column temperature and a 1 mL/min flow rate, with the ratio of solvent A (methanol):solvent B (water) ¼ as 28:72 (*v*/*v*), and the wavelength was 214 nm [15]. The standard curve was y = 131.90764x − 3.04545 (R^2^ = 0.9984), and the content of amygdalin was calculated.

Determination of hydrocyanic acid: The pH of the solution was adjusted using acetic acid and sodium hydroxide, followed by the addition of chloramine T for the reaction. Subsequently, isonicotinic acid-barbituric acid was introduced to develop the color, and the absorbance was measured at a wavelength of 600 nm [16]. The standard curve was y = 0.85882x − 0.00781 (R^2^ = 0.9991), and the content of hydrocyanic acid was calculated.

### 2.4. Process Optimization of Degrading Amygdalin into Benzaldehyde by Response Surface Methodology

Based on the results of single-factor tests, three independent variables were set (X_1_-time; X_2_-temperature; X_3_-pH) with the content of benzaldehyde as the dependent variable, and the Box–Behnken design of Response Surface Methodology (RSM) was employed for optimization. There were a total of 15 test points, including 3 axial points for assessing experimental errors, and the remaining 12 sets were used as the analysis points. Table 1 displays the encoded variables concerning the experimental factors and levels. Design Expert 8.0.6 software was used to design the experiments and calculate the data.

### 2.5. Preparation of the bz-β-CD

We referred to Abarca’s encapsulation preparation process with slight modifications [17]. Benzaldehyde was dissolved in anhydrous ethanol at a volume ratio of 1:30 (mL/mL). The ethanol solution of benzaldehyde was added to the aqueous β-CD solution for embedding, and the mixture was left for 2 h. Afterwards, it was removed and allowed to cool to room temperature, and then it was refrigerated at 4 °C overnight. The final product, bz-β-CD, was obtained through filtration and drying.

### 2.6. Characterization of the bz-β-CD Encapsulation Effect

#### 2.6.1. Determination of Encapsulation Rate

The rate (%) of the encapsulation, indicating the amount of benzaldehyde encapsulated in the β-CD cavity, was determined using the Heydari method [18]. For the determination of the total oil content of microcapsules, 0.1 g bz-β-CD was weighed out, fully immersed in 20 mL anhydrous ethanol, ultrasonicated at 300 W, 59 HZ for 30 min, and allowed to stand for 1 h. The content of benzaldehyde in ethanol was then determined by the method shown in Section 2.3.

According to the measured surface oil and the total oil content, the embedding rate was calculated using Equation (1).

Encapsulation rate = (*V*_1_ − *V*_2_)/*V*_1_ × 100% (1)

Note—*V*_1_: the volume of total oil in microcapsules (mL). *V*_2_: the surface oil volume of microcapsules (mL).

#### 2.6.2. The Effects of Different Wall–Core Ratios on Encapsulation Rate

The bz-β-CD was prepared with wall–core ratios (mL/g) of β-CD to benzaldehyde of 10:1, 5:1, 10:3, 5:2, and 2:1. The total oil and surface oil volume of the encapsulation were measured and the encapsulation rate was determined according to the method shown in Section 2.6.1.

#### 2.6.3. Fourier Transform Infrared Spectroscopy Analysis (FI-IR)

The potassium bromide and the sample were dried separately and then mixed for grinding. Subsequently, they were pressed into tablets and scanned in the range of 400–4000 cm^−1^ using a VERTEX 70v FT-IR Spectrometer (BRUKER, Karlsruhe, Germany) The number of scans was 16 and the resolution was 4 cm^–1^.

#### 2.6.4. Thermo-Gravimetric Analysis (TGA)

Thermogravimetric analysis was performed using the thermal analysis equipment. The flow rate of N_2_ gas was 20 mL/min, the rate of temperature increase was 10 °C/min, and the maximum temperature was 650 °C. In this way, the final thermal weight loss curve was obtained.

#### 2.6.5. Particle Size Distribution Analysis

The particle size and distribution of the bz-β-CD were determined by laser diffraction analysis using an LS13320 laser particle size distribution instrument. Each group of samples to be tested was repeatedly measured three times, and the D_10_, D_50_, D_90_ and Span of the encapsulation were measured. The relationship between the four is expressed by the following Equation (2).

Span = (*D*_90_ − *D*_10_)/*D*_50_ (2)

Note—Span: the particle size distribution range of encapsulation. *D*_90_: the cumulative distribution of particles of 90%. *D*_10_: the cumulative distribution of particles is 10% of the particle size. *D*_50_: the cumulative distribution of particles is 50% of the particle size used to represent the average particle size of encapsulation.

#### 2.6.6. Release Kinetics Analysis

The bz-β-CD was allowed to stand at 5 °C, 30 °C, and 55 °C for a specific duration. Samples were taken on days 0, 1, 2, 3, 4 and 5 to determine the total oil content via the method in Section 2.6.1, and we calculated the change in the benzaldehyde retention rate of encapsulation using the following Equation (3):

Retention (%) = *V*_4_/*V*_3_ × 100% (3)

Note—*V*_3_: The initial volume of bz-β-CD (mL). *V*_4_: The current volume of bz-β-CD (mL).

The release kinetics of bz-β-CD have been studied by fitting the zero-order kinetics, first-order kinetics and Avrami equation according to the retention rate, using the method of Ren [19].

#### 2.6.7. Inhibition Experiment of Mycelial Growth of *Botrytis cinerea*

Following the method outlined by Edwards, the inhibitory effects of benzaldehyde and bz-β-CD on *Botrytis cinerea* were determined using the mycelial growth rate method [20]. The colony growth diameter was measured using the cross method, and the growth inhibition rate of the mycelium was calculated. Then, the drug concentration was converted into the logarithm of the concentration, and the mycelium growth inhibition rate was converted into the inhibition rate probability value. The regression equation (y = aX + b) and correlation coefficient (R^2^) were obtained, and the effective bacteriostatic concentration (EC_50_) was calculated.

### 2.7. Molecular Simulation

#### 2.7.1. Molecular Docking Simulation

Molecular docking of the β-CD and benzaldehyde molecules was performed using AutoDockTools-1.5.6 software, applying the empirical free energy function and Lamarckian genetic algorithm [21]. The initial structure of the β-CD molecule was obtained from the Cambridge crystal database (CCDC, https://www.ccdc.cam.ac.uk/ (accessed on 9 June 2023)) (CCDC ID: 1235577). Likewise, the initial structure of the benzaldehyde molecule was derived from the ZINC database (http://zinc.docking.org/ (accessed on 9 June 2023)) Each docking process consisted of 50 independent dockings, and the conformation with the lowest docking energy was selected based on energy magnitude.

#### 2.7.2. Molecular Dynamics Simulation

Molecular dynamics simulations were undertaken via the previously reported methods, with some modifications made [22]. The initial conformation of bz-β-CD was taken from the previous docking results, and the topology was created based on the available GROMACS database. GROMACS 2020.6 software was used to simulate β-CD and bz for 50 ns.

### 2.8. Statistical Analysis

All the above measurements were repeated in triplicate, and the results have been expressed as mean ± standard deviation. The significance of the data was analyzed using SPSS 25 software. *p* < 0.05 was considered as indicating a significant correlation, and *p* < 0.01 was considered as indicating an extremely significant correlation. In addition, Origin2021 was used to draw the relevant charts. SPSS was used for the significance analysis.

## 3. Results and Discussion

### 3.1. Optimization of Benzaldehyde Preparation Process

The results of the test are shown in Table 2, and the effects of the three factors on the content of benzaldehyde have been inferred from the results of the following quadratic polynomial regression equation. The independent variables and the test variables were correlated using the following second-order polynomial equation:

y = 2.12 − 0.11 × X_1_ − 0.15 × X_2_ − 1.86 × 10^−3^ × X_3_ − 0.014 × X_1_ × X_2_ + 0.13 × X_1_ × X_3_ − 0.041 × X_2_ × X_3_ − 0.18 × X_1_^2^ − 0.42 × X_2_^2^ − 0.14 × X_3_ (4)

**Figure 1:**
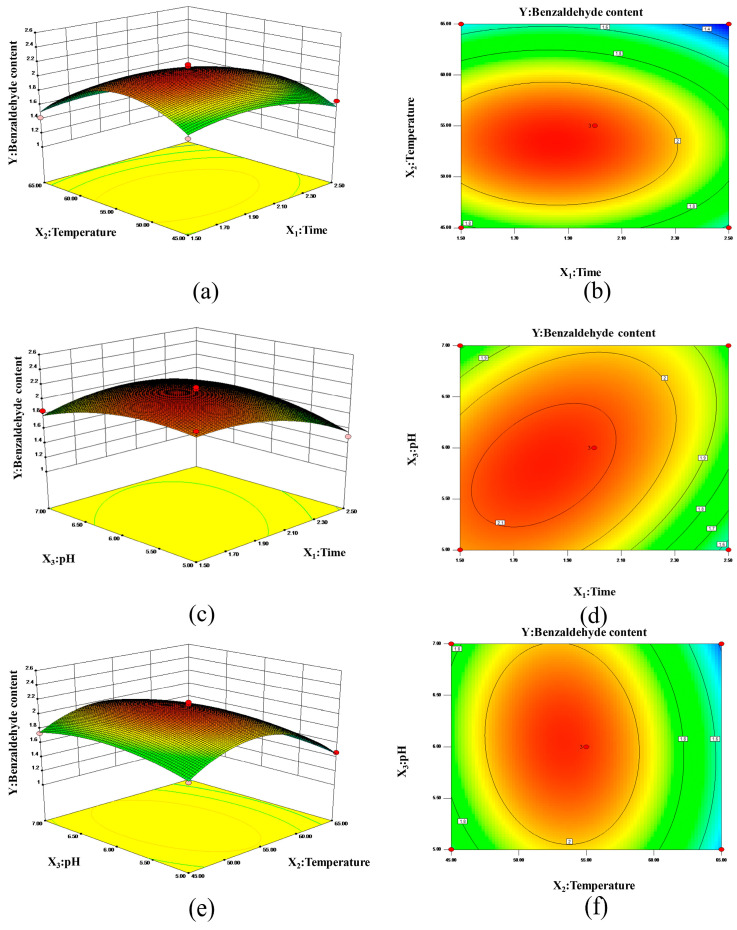
Response surface plots and contour plots of preparation process of benzaldehyde. Note: (**a**,**c**,**e**): Response surface plots of the effects of temperature and time, pH and time, pH and temperature on the content of benzaldehyde, respectively. (**b**,**d**,**f**): Contour plots of the effects of temperature and time, pH and time, pH and temperature on the content of benzaldehyde, respectively. The colors represent the size of the result.

The results of the analysis of variance, performed via a regression model, are shown in Table 3. The F value of the model is 13.95, the *p* value is 0.0049, and the *p* value of the mismatch term is 0.2395, indicating that the model is significant and can be used for optimization. At the same time, it can be seen from Table 3 that the linear coefficient (X_1_, X_2_) and cross coefficient (X_1_X_3_) are significant at the *p* < 0.05 level. The three-dimensional response surface and contours of benzaldehyde are shown in Figure 1a,c,e. In general, an elliptical contour represents a significant interaction between independent variables, while a circular interaction is not significant [5]. From Table 3 and Figure 1b,d,f, we can infer that the interaction between X_1_ and X_3_ is significant, while the interaction between X_1_X_2_ and X_2_X_3_ is not significant. This indicates that the effect of time and pH on the production of benzaldehyde involves a certain interaction. According to the predictions of the quadratic regression model, the optimum parameters for benzaldehyde production are: time 1.82 h, temperature 53.34 °C, pH 5.84 and benzaldehyde content 2.1539 g/L. For industrial applications, the optimum formula can be modified as: time 1.8 h, temperature 53 °C, pH 5.8 and benzaldehyde content 2.1521 g/L. The relative error between the predicted value and the measured value is only 0.8%, indicating that the model has high accuracy and can simulate the actual situation well.

After the addition of the apricot kernel, the amygdalin in the apricot kernel-debitterizing wastewater decreased from 14.6 to 1.98 g/L, representing a degradation rate of 86.43%. The content of benzaldehyde in the final product was 198 g/L, and the cyanide content was 4.856 × 10^−6^ g/L. These results indicate that this method enables environmentally friendly, rapid, and mild amygdalin degradation. The benzaldehyde obtained through this method exhibited high purity, while the cyanide content remained low. The cyanide produced by the degradation of amygdalin was enriched in the distilled liquid through steam distillation, facilitating the debitterizing wastewater treatment. Overall, these findings suggest that this method is a viable and environmentally friendly approach to benzaldehyde preparation.

### 3.2. Characterization of Benzaldehyde Encapsulation by β-CD

#### 3.2.1. The Effects of Different Wall–Core Ratios on the Encapsulation Rate of bz-β-CD

**Figure 2:**
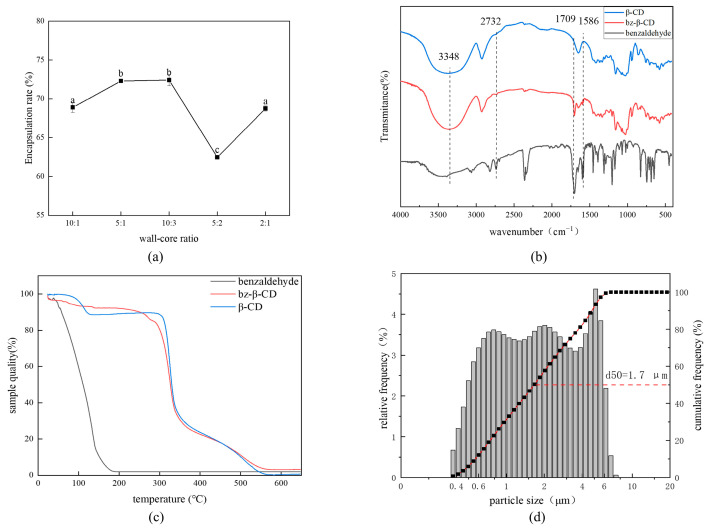
Characterization of bz-β-CD. Note: (**a**) Effects of different wall–core ratios on encapsulation rate; a–c in Figure 2a are the results of LSD test. Different letters indicate significant differences in results; (**b**) FTIR spectra of β-CD, benzaldehyde, and bz-β-CD; (**c**) TGA curve of β-CD, benzaldehyde, and bz-β-CD; (**d**) Particle size distribution of bz-β-CD.

It can be seen from Figure 2a that the encapsulation rate increased first, and then decreased, with the rise in the wall–core ratio. The wall–core ratios of 5:1 and 10:3 (mL/g) exhibited significantly higher encapsulation rates than the other three ratios. Due to the limited capacity of β-CD to encapsulate benzaldehyde, the excess benzaldehyde will not be encapsulated, so a high wall–core ratio results in a decrease in the encapsulation rate. Therefore, in the subsequent experiments, a wall–core ratio of 5:1 (mL/g) was chosen as the encapsulation condition for benzaldehyde.

#### 3.2.2. Fourier Transform Infrared Spectroscopy Analysis of bz-β-CD

The infrared spectrum, presented in Figure 2b, reveals several notable absorption peaks. Specifically, at 2732 cm^−1^, 1709 cm^−1^, and 1586 cm^−1^, distinct absorption peaks correspond to the C–H stretching of the benzaldehyde aldehyde group, the C=O stretching of the benzaldehyde aldehyde group, and the benzaldehyde benzene ring, respectively [23]. The absorption peaks of bz-β-CD at 2732 cm^−1^ and 1709 cm^−1^ were different from those of β-CD, but the same as those of benzaldehyde, indicating the encapsulation of benzaldehyde by β-CD. Additionally, bz-β-CD also showed different absorption peaks at 1586 cm^−1^ from β-CD, because β-CD lacked a benzene ring and the encapsulated bz-β-CD contained a benzene ring. However, the absorption peaks of the aldehyde C–H bond and the benzene ring of bz-β-CD are not obvious, which may be due to the masking or inhibitory effects of the β-CD cavity on the absorption vibration. Qiao observed similar phenomena in the infrared spectrum of the HP-β-CD–astaxanthin ester inclusion complex, whereby some characteristic bands belonging to the six-membered ring of astaxanthin ester were missing, presumably because the six-membered ring entered the HP-β-CD cavity and formed a hydrogen bond with -OH on the cavity [24]. In conclusion, it was proven that benzaldehyde was encapsulated in β-CD.

#### 3.2.3. Thermogravimetric Analysis of β-CD, Benzaldehyde, and bz-β-CD

According to the TGA curve shown in Figure 2c, benzaldehyde underwent a weight loss of up to 98.4% in the temperature range of 40 °C to 180 °C, indicating that the thermal stability of benzaldehyde is poor. In contrast, the initial weight loss of bz-β-CD was only 8.74% at 25 °C to 143 °C, associated with a loss of water during the heating process. The second stage of weight loss occurred between 260 °C and 370 °C, with a notable weight reduction of 72.9%, related to the thermal decomposition of the composite in this stage. In the temperature range of 200 °C to 300 °C, bz-β-CD underwent a degree of weight loss different from β-CD, due to the volatilization of benzaldehyde in bz-β-CD. In the case of complexation, the interaction between the host and guest enhances the stability of typically volatile guest molecules within the inclusion complex. After the formation of bz-β-CD, the mass loss of benzaldehyde occurs at temperatures ranging from 200 °C to 300 °C, encapsulating the benzaldehyde and enabling it to endure higher temperatures. In summary, the thermal stability of benzaldehyde is significantly enhanced after encapsulation by β-CD. Celebioglu reported that the weight loss of free eugenol was completed at 50–190 °C, and the inclusion complex had two weight loss processes, namely, the dehydration process before 130 °C and the main degradation process at 330 °C, indicating that the thermal stability of eugenol was significantly improved after encapsulation with γ-cyclodextrin [25]. The change in thermal stability after encapsulation was consistent with this research. This phenomenon also proves that the encapsulation of β-CD enhances thermal stability, and β-CD can be employed for encapsulating benzaldehyde.

#### 3.2.4. Particle Size Analysis of bz-β-CD

As shown in Figure 2d, the particle size distribution of bz-β-CD is mainly between 0.4 and 7.42 μm, with a capsule size of 1.707 μm. The span measures 2.72 μm, indicating a relatively narrow particle size distribution. These data collectively indicate that the prepared encapsulation exhibited minimal variations in particle size and a tightly concentrated distribution. These characteristics suggest that bz-β-CD did not undergo significant polymerization.

#### 3.2.5. Effects of Different Temperatures on the Release Kinetics of bz-β-CD

**Figure 3:**
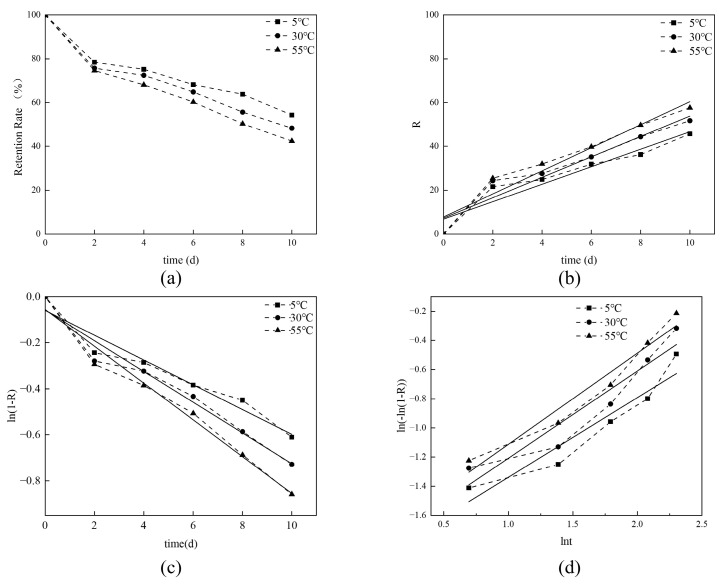
Release kinetics analysis of bz-β-CD. (**a**) Release curve of bz-β-CD at three different temperatures; (**b**) zero-order fitting; (**c**) first-order fitting; (**d**) Avrami’s fitting.

The fitting curve of encapsulation release kinetics is shown in Figure 3, and the specific fitting equation and R^2^ are shown in Table 4. Under conditions of 5 °C, 35 °C, and 55 °C, the R^2^ of each regression equation was above 0.9, indicating that Avrami’s equation had a good fit to the release process of benzaldehyde at different temperatures. The values of the release mechanism parameter “*n*” for the encapsulation at 5 °C, 35 °C, and 55 °C were determined as 0.5467, 0.5982, and 0.6252, respectively. These values fall within the range of 0.54 to 1.00, signifying that the release reaction kinetics align with a regime somewhere between diffusion-limited kinetics and first-order reaction kinetics. Compared with zero-order kinetic fitting, first-order kinetic fitting showed excellent fitting, and was able to more accurately represent the release behavior of benzaldehyde.

At 5 °C, 35 °C and 55 °C, the release rate constants (k) calculated by Avrami’s equation were 0.1518, 0.1643 and 0.1760, respectively, which increased with the increase in temperature. This result indicated that benzaldehyde is indeed encapsulated within β-CD, and temperature exerts a pronounced influence on its release characteristics. Consequently, it is advisable to store bz-β-CD at lower temperatures for enhanced control over benzaldehyde release.

#### 3.2.6. Effects of Different Concentrations of bz and bz-β-CD on Antifungal Activity of *Botrytis cinerea*

**Figure 4:**
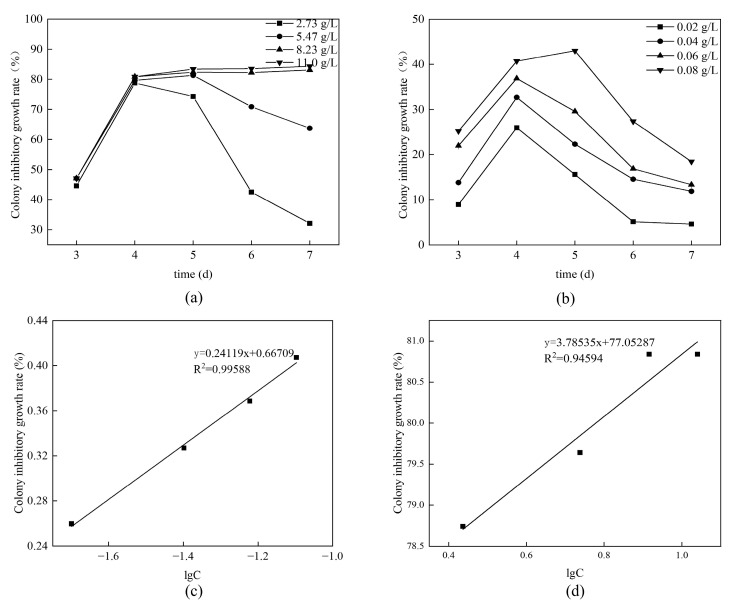
Antibacterial effects of benzaldehyde and bz-β-CD. (**a**) The effects of different concentrations of benzaldehyde on the inhibition of *Botrytis cinerea* colony growth. (**b**) The effects of different concentrations of bz-β-CD on the inhibition of *Botrytis cinerea* colony growth. (**c**) Fitting curves of benzaldehyde concentration and colony growth rate inhibition. (**d**) Fitting curves of bz-β-CD concentration and colony growth rate inhibition.

As shown in Figure 4a,b, bz-β-CD and benzaldehyde have different capacities for inhibiting the colony growth of *Botrytis cinerea*. It is shown that the inhibition rate of mycelial growth follows a concentration-dependent pattern, with antibacterial efficacy increasing as concentration rises. The effective inhibitory concentrations (EC_50_) were calculated to be 0.2029 μg/mL for benzaldehyde and 0.1929 μg/mL for bz-β-CD, based on linear regression equations derived from different concentrations and their corresponding mycelial growth inhibition rates, as presented in Figure 4b,d. Because the effective inhibitory concentration was inferred from the bacteriostatic diameter on the fourth day of determination, it was speculated that the EC50 of benzaldehyde was lower than that of bz-β-CD due to the volatility of benzaldehyde. As shown in Figure 4a,b, the relative inhibition rate of benzaldehyde increased rapidly and then decreased rapidly, while the relative inhibition rate of bz-β-CD increased rapidly and then decreased slowly. This result indicates that the sustained release effect induced by encapsulation successfully prolonged the antibacterial effect of benzaldehyde on *Botrytis cinerea*, thus achieving a long-lasting antibacterial impact.

### 3.3. The Analysis of the Encapsulation Mechanism of Benzaldehyde and β-CD

#### 3.3.1. Analysis of Molecular Docking Results of Benzaldehyde and β-CD

**Figure 5:**
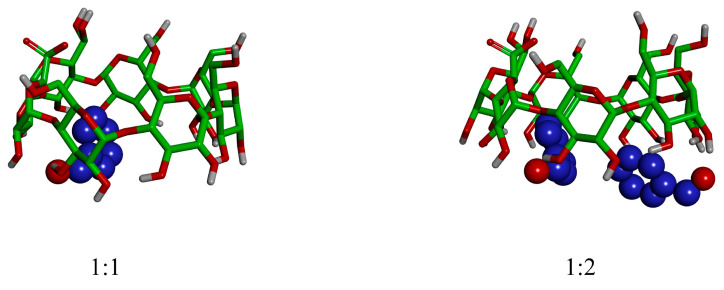
Molecular docking conformations with different molar ratios of β-CD to benzaldehyde. The colors are used to distinguish between different atoms and molecules.

As shown in Figure 5, the docking results show that the host β-CD molecule and the guest benzaldehyde molecule recognized one-to-one molecules without the limitation of a steric hindrance effect, indicating that the sizes of the host and the guest molecules were well-matched. Subsequently, the molecular docking results, with a molar ratio of 1:2 between the host β-CD molecule and the guest benzaldehyde molecule, indicate that there is a guest benzaldehyde molecule that does not enter the cavity of the β-CD molecule. This benzaldehyde molecule did not fully interact with the β-CD cavity and remained in a free state, indicating that this conformation was not stable. The difference in the docking state was attributed to the limited cavity size of the β-CD molecule, which made it impossible for it to accommodate two aromatic molecules at the same time, contrary to the principle of host–guest molecular size matching [26]. Molecular docking results for a molar ratio of 2:1 between the host β-CD molecule and guest benzaldehyde molecule have not been discussed because, as Ohga pointed out, the dimer conformation of β-CD is random and unstable [27]. It is shown in the conformation analysis that the benzene ring of benzaldehyde was encapsulated in β-CD, confirming that benzaldehyde was encapsulated by β-CD through the encapsulation process shown in Section 2.5, which is consistent with the speculation that the encapsulation of the benzene ring’s benzaldehyde (as analyzed in Section 3.2.2) resulted in an unobvious absorption peak. However, the optimal wall–core ratio for encapsulation, as determined in the experiment, was 5:1, which is not consistent with the ratio obtained by molecular docking. This discrepancy could be attributed to the volatilization of benzaldehyde during the encapsulation process, or the incomplete reaction between benzaldehyde and β-CD. Subsequently, the further optimization of the encapsulation process is warranted. The potential optimization of this process is a prospect for future research.

#### 3.3.2. The Impacts of Temporal Variations on Conformation

**Figure 6:**
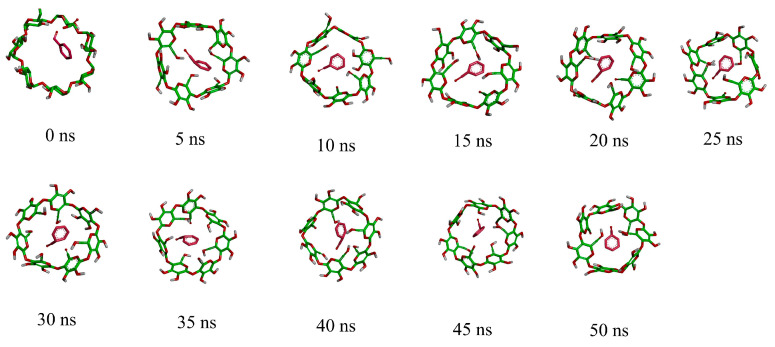
The conformation changes of bz-β-CD with time variations. The colors are used to distinguish between different atoms and molecules.

The process of inclusion is inherently dynamic, leading to continuous changes in the conformation of the inclusion complex over time. As time progresses, intermolecular interaction forces tend to reach equilibrium, the system’s energy stabilizes, and molecular conformations attain a steady state. In Figure 6, the dynamic evolution of the bz-β-CD inclusion complex is depicted over intervals of 5 ns, transitioning from its initial state at 0 ns to a stable state at 50 ns.

During the simulation, the benzaldehyde molecule enters the β-CD cavity through the larger opening with only minor positional changes, and the ring structure of benzaldehyde consistently remains within the cavity. This stability is attributed to the planar orientation of the six carbon atoms in the benzene ring, which provides a higher rigidity, resulting in the ring plane not bending or folding to accommodate the narrow part of the cavity.

#### 3.3.3. Root Mean Square Deviation Analysis

**Figure 7:**
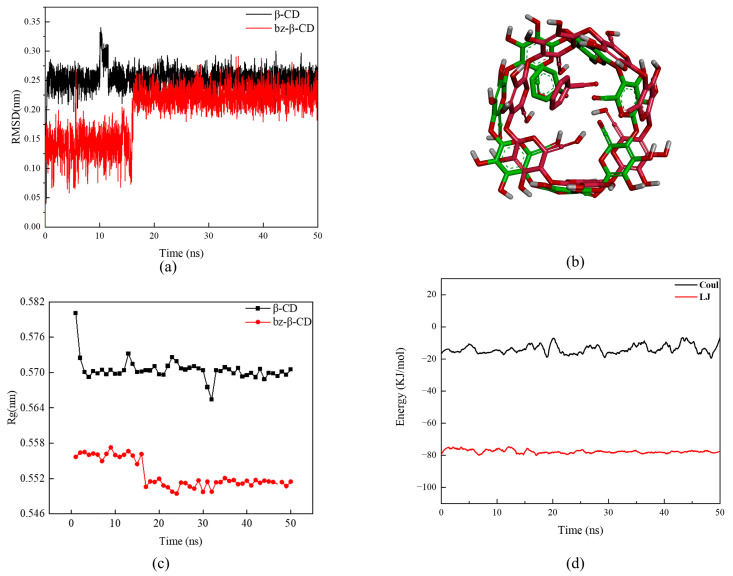
The changes with time in the simulation process of bz-β-CD. (**a**) The change in root mean square deviation in the simulation process; (**b**) the change in bz-β-CD molecular conformation before and after mutation (green: before mutation, red: after mutation); (**c**) the radius of gyration of β-CD and bz-β-CD; (**d**) MMPBSA analysis of bz-β-CD.

Root mean square deviation (RMSD) quantifies the variance in molecular structure relative to the initial structure throughout the simulation, offering insights into conformational convergence and stability. When the RMSD fluctuates within a small range, it has been considered that the structural convergence of the molecule will be stable [28]. During the initial 0–15 ns interval, both β-CD and benzaldehyde molecules underwent significant positional shifts and structural distortions, resulting in substantial RMSD fluctuations (0.14 ± 0.02 nm). However, post-mutation, the molecular RMSD values stabilized (0.22 ± 0.01 nm). The RMSD value of bz-β-CD is smaller than that of β-CD, which is because the introduction of a bz molecule into the inclusion complex makes the structure of β-CD more stable. From Figure 7b, it can be seen that the structure of β-CD changed, with alterations in the angles of specific C–O–C bonds, thereby reducing the size of the hydrophobic cavity.

#### 3.3.4. Radius of Gyration Analysis of bz-β-CD

As shown in Figure 7c, the radius of gyration (Rg) of bz-β-CD is notably smaller than that of free β-CD, indicating that the structure of bz-β-CD tends to shrink, and the structure is more compact than that of free β-CD due to the interaction between benzaldehyde and β-CD molecules in the inclusion complex. The change in the Rg value of the free-state guest benzaldehyde molecule is due to the conformational rearrangement of its own structure. The differences in te Rg values between β-CD and bz-β-CD indicate that β-CD has undergone structural changes during its interaction with the benzaldehyde molecule [21].

The Rg of bz-β-CD underwent a significant change between 15 and 17 ns, which was consistent with the structural change in β-CD at this stage, as highlighted in the RMSD analysis. Post-mutation, the Rg values remain stable, ranging between 0.551 and 0.549 nm, with minimal fluctuations (<0.01 nm) indicating that the structure of bz-β-CD has reached a stable state. The Rg value of bz-β-CD fluctuates greatly with time and gradually decreases, which may be attributed to the fact that the interaction of the bz-β-CD structure tends to stabilize over time.

#### 3.3.5. Interaction Energy Analysis of bz-β-CD

As shown in Table 5, the entry of benzaldehyde molecules into the cavity of β-CD is a result of the intermolecular interaction, featuring a Coulomb interaction and a van der Waals interaction. Benzaldehyde is a hydrophobic molecule, and β-CD also has a hydrophobic cavity, so the solvent energy is positive in this context. The hydroxyl oxygen atom and the glycosidic bond oxygen atom of β-CD have strong electronegativity, inducing dipole formation during molecular interactions, thus facilitating van der Waals interactions. Additionally, due to the strong electronegativity of the hydroxyl oxygen atom, there is a potential difference between the hydroxyl group and the surrounding molecules, and so there is a Coulomb interaction between the molecules.

It can be seen from Figure 7d that the Lennard–Jones (LJ) potential and Coul potential are relatively stable during the interaction. The Coul potential fluctuates around −18 kJ/mol, indicating that there is electrostatic attraction and repulsion at play in the molecular interaction, and this interaction is very weak. The LJ potential between benzaldehyde molecules is −80 kJ/mol, consistently surpassing the Coul potential, indicating that the primary driving force of the entry and retention of benzaldehyde molecules within the cavity is van der Waals interactions. After calculation, we found that the overall binding free energy of bz-β-CD is negative, indicating that the binding can proceed spontaneously, which agrees with the results of Lu’s research [26].

## 4. Conclusions

In summary, the amygdalin present in apricot kernel-debitterizing wastewater can be degraded in an environmentally friendly way to produce benzaldehyde, and the degradation rate of amygdalin can be increased by 86.43% with the optimization of the degrading parameters, such as the reaction time, temperature, and pH. Besides this, the bz-β-CD possessed different properties from the benzaldehyde itself, and the encapsulation of benzaldehyde with β-CD facilitated noticeable enhancements in the inhibition effect and thermal stability. Additionally, the encapsulation of benzaldehyde with β-CD is induced by negative binding energy, itself mainly affected by the van der Waals force and Coulomb force, and the encapsulated complex shows less fluctuation and structural change compared to the benzaldehyde itself. In a word, the apricot kernel-debitterizing wastewater can serve as a raw material for benzaldehyde production, and the β-CD can effectively encapsulate the benzaldehyde, thus broadening the application of benzaldehyde. In the future, apricot kernel-debitterizing wastewater will no longer be considered as waste by food companies; instead, it will be utilized as a valuable raw material. Additionally, benzaldehyde microcapsules can find applications in food packaging materials and food storage processes. However, methods for industrializing the amygdalin degradation process and the effects of bz-β-CD on other fungi should be further studied.

## Figures and Tables

**Table 1:** Factors and their levels of BBD for the preparation process of benzaldehyde.

Independent Variables	Factor		
	−1	0	1
X_1_—Time (h)	1.5	2	2.5
X_2_—Temperature (°C)	65	55	45
X_3_—pH	5	6	7

**Table 2:** Box–Behnken experiment results for the preparation of benzaldehyde.

Test	X_1_—Time (h)	X_2_—Temperature (°C)	X_3_—pH	Y—Benzaldehyde (g/L)
1	2	45	5	1.674874309
2	1.5	45	6	1.726983809
3	2.5	45	6	1.662509763
4	2	45	7	1.740413837
5	2.5	55	5	1.496666262
6	1.5	55	5	2.11229181
7	2	55	6	2.169431375
8	2	55	6	2.14237309
9	2	55	6	2.053541632
10	2.5	55	7	1.757337655
11	1.5	55	7	1.848597421
12	2	65	5	1.469880542
13	2.5	65	6	1.302500764
14	1.5	65	6	1.397502106
15	2	65	7	1.514259103

**Table 3:** Variance analysis of regression equation.

Source	SS	df	MS	F Value	*p* Value	
Model	1.11	9	0.12	13.95	0.0049	**
X_1_	0.099	1	0.099	11.27	0.0202	*
X_2_	0.18	1	0.18	20.02	0.0066	**
X_3_	2.781 × 10^−5^	1	2.781 × 10^−5^	3.157 × 10^−3^	0.9574	
X_1_X_2_	7.647 × 10^−4^	1	7.647 × 10^−4^	0.087	0.7801	
X_1_X_3_	0.069	1	0.069	7.80	0.0383	*
X_2_X_3_	6.618 × 10^−3^	1	6.618 × 10^−3^	0.75	0.4258	
X_1_^2^	0.12	1	0.12	13.13	0.0152	
X_2_^2^	0.64	1	0.64	72.60	0.0004	
X_3_^2^	0.073	1	0.073	8.34	0.0343	
Residual	0.044	5	8.811 × 10^−3^			
Lack of Fit	0.037	3	0.012	3.33	0.2395	
Pure Error	7.351 × 10^−3^	2	3.676 × 10^−3^			
Cor Total	1.15	14				

Note: * represents a significant difference (*p* < 0.05), ** represents a highly significant difference (*p* < 0.01).

**Table 4:** Release kinetics parameter of bz-β-CD.

	Temperature (°C)	Fitting Equation	R^2^
Avrami’s fitting	5	y = 0.54665x − 1.88522	0.91091
	30	y = 0.59824x − 1.80598	0.90536
	55	y = 0.62519x − 1.73751	0.95358
Zero-order fitting	5	y = 3.98832x + 6.76983	0.91297
	30	y = 4.65976x + 7.24012	0.92636
	55	y = 5.26538x + 7.75528	0.93743
First-order fitting	5	y = −0.5382x − 0.0598	0.94713
	30	y = −0.6676x − 0.05795	0.96374
	55	y = −0.7991x − 0.05568	0.97764

**Table 5:** MMPBSA analysis of bz-β-CD.

Energy	Complex
Van der Waals energy (KJ/mol)	−77.278
Electrostatic energy (KJ/mol)	−13.282
Polar solvation energy (KJ/mol)	39.041
Nonpolar solvation Energy (KJ/mol)	−9.829
Total binding energy (KJ/mol)	−61.348
T∆S (KJ/mol)	9.615
Total binding free energy (KJ/mol)	−51.733

## Data Availability

Data are contained within the article.
